# Macrophage activation marker sCD163 is associated with liver injury and hepatic insulin resistance in obese patients before and after Roux‐en‐Y gastric bypass

**DOI:** 10.14814/phy2.15157

**Published:** 2022-01-18

**Authors:** Konstantin Kazankov, Kirstine Nyvold Bojsen‐Møller, Holger Jon Møller, Sten Madsbad, Henning Grønbæk

**Affiliations:** ^1^ Department of Hepatology and Gastroenterology Aarhus University Hospital Aarhus Denmark; ^2^ Institute for Liver and Digestive Health University College London London United Kingdom; ^3^ Department of Endocrinology Copenhagen University Hospital Hvidovre Hvidovre Denmark; ^4^ Department of Clinical Biochemistry Aarhus University Hospital Aarhus Denmark; ^5^ Novo Nordisk Foundation Center for Basic Metabolic Research University of Copenhagen Copenhagen Denmark

**Keywords:** adiposity, bariatric surgery, diabetes, Kupffer cells

## Abstract

**Background:**

Macrophages are associated with metabolic complications to obesity including fatty liver disease and impaired hepatic and muscle insulin sensitivity (IS). Bariatric surgery induces weight loss and improves IS. We investigated associations between the macrophage activation marker soluble (s)CD163, alanine‐aminotransferase (ALT), and IS before and after Roux‐en‐Y Gastric Bypass (RYGB).

**Methods:**

We analyzed sCD163 from 10 type 2 diabetes (T2D) and 10 obese patients with normal glucose tolerance (NGT) undergoing RYGB for associations with hepatic, adipose tissue, and muscle IS and ALT after 1‐week, 3, and 12 months postoperatively. IS was evaluated by hyperinsulinemic‐euglycemic clamp in combination with glucose tracer technique.

**Results:**

Preoperative sCD163 correlated with ALT (*r* = 0.58, *p* = 0.007) and tended to associate inversely with hepatic (*r* = −0.39, *p* = 0.1) and adipose tissue (*r* = −0.39, *p* = 0.09), but not muscle IS. Following RYGB, sCD163 decreased significantly in all patients. The decrease in sCD163 during the first 3 months correlated inversely with the improvement of hepatic IS (*r* = −0.65, *p* = 0.01) and tended to be associated with changes in muscle IS (*r* = −0.45, *p* = 0.09). After 3 months sCD163 remained associated with ALT (*r* = 0.75, *p* < 0.001) and inversely with hepatic IS (*r* = −0.39, *p* = 0.1), but not muscle or adipose tissue IS. One year after RYGB, sCD163 correlated with ALT (*r* = 0.61, *p* = 0.007), but not with hepatic, adipose tissue, or muscle IS.

**Conclusion:**

Macrophage activation is associated with liver injury and hepatic IS in obese patients. Improvements in these measures correlate during the first 3 months following RYGB, supporting a link between macrophages and hepatic IS in severe obesity and diabetes.

## Graphical abstract

Macrophage activation assessed by soluble CD163 is associated with hepatic insulin resistance in patients with severe obesity and its improvement after Roux‐en‐Y gastric bypass and weight loss.

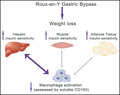

## 1 INTRODUCTION

Obesity is a major healthcare issue closely associated with the metabolic syndrome, impaired insulin sensitivity (IS), and type 2 diabetes (T2D), as well as metabolic‐associated fatty liver disease (MAFLD). Bariatric surgery is an effective treatment of severe obesity that induces sustained major weight loss (~40 kg or 15 BMI units) and favorable changes in IS leading to improved glycemic control or remission of T2D (Nguyen & Varela, 2017). Following Roux‐en‐Y gastric bypass (RYGB), hepatic IS improves rapidly, likely mediated by immediate calorie restriction and early decrease in liver fat. On the other hand, improvement in muscle IS occurs later, probably attributable to the subsequent weight loss (Bojsen‐Moller et al., 2014).

Macrophages play an important role in metabolic derangement in obesity including impaired IS and T2D, as well as MAFLD (Fuentes et al., 2010; Kazankov et al., 2019). Consistently, the specific marker of macrophage activation soluble CD163 (sCD163) is associated with the presence and severity of these conditions (Fjeldborg et al., 2013; Kazankov et al., 2016; Parkner et al., 2012). Furthermore, macrophage activation assessed by sCD163 declines after weight loss induced by diet, exercise, and bariatric surgery in association with improvement in MAFLD features and the homeostatic model assessment of insulin resistance (HOMA‐IR) (Kazankov et al., 2015; Rodgaard‐Hansen et al., 2017). However, HOMA‐IR is a surrogate marker of primarily hepatic insulin resistance (IR) and does not always correspond to the gold standard methods such as for example, hyperinsulinemic‐euglycemic clamp (HEC), especially in patients undergoing RYGB (Bojsen‐Moller et al., 2017).

In this study, we assessed macrophage activation using sCD163 in an established cohort of obese patients with T2D and patients with obesity and normal glucose tolerance (NGT) undergoing RYGB. The concentrations of sCD163 at baseline and different time points after RYGB were tested for associations with hepatic, adipose tissue, and muscle IS measured by state‐of‐the‐art methodology. We hypothesized cross‐sectional and dynamic associations of sCD163 with IS and liver enzymes.

## 2 METHODS

### 2.1 Study design and participants

We studied 10 obese patients with T2D (4 men, 6 women) and 10 patients with obesity and NGT (3 men, 7 women) who underwent laparoscopic RYGB at Hvidovre Hospital (Hvidovre, Denmark). The study population and design have been described in detail in previously published studies (Albers et al., 2015; Bojsen‐Moller et al., 2014, 2017). In brief, the patients were examined prior to surgery, and 1‐week, 3 months, and 12 months post‐surgery. Prior to study enrollment, all participants had completed a preoperative diet‐induced weight loss of at least 8% as required by Danish health authorities (https://www.sst.dk/‐/media/Udgivelser/2017/Retningslinjer‐for‐visitation‐til‐kirurgisk‐behandling‐af‐sv%C3%A6r‐fedme‐190517.ashx?la=da&hash=D949F5554592C0593C734ECDD420016B13EE39ED). All tests were done after an overnight fast >10 h and after discontinuation of antidiabetic medication for at least 3 days. In the NGT group, all patients had a 2‐hour P‐glucose concentration after an oral glucose tolerance test <7.8 mmol/L and HbA1c <6% (42 mmol/mol).

Written informed consent was obtained from all participants and the study was approved by the Municipal Ethical Committee of the Capital region of Denmark (protocol number H‐3‐2010‐041) in accordance with the Declaration of Helsinki and by the Danish Data Protection Agency and registered at www.ClinicalTrials.gov (NCT 01202526).

### 2.2 Hyperinsulinemic‐euglycemic clamp

The HEC has been described in details in previous publications (Albers et al., 2015; Bojsen‐Moller et al., 2014, 2017). In short, the HEC was combined with [6,6‐^2^H_2_]‐glucose (99 atom percent enrichment; Cambridge Isotope Laboratories, Andover, MA) administered for 120 min prior to and during the clamp. The clamp was initiated with a 4‐hour primed continuous insulin infusion of 40 mU/m^2^/min (Actrapid; Novo Nordisk, Bagsværd, Denmark) combined with a variable infusion of 20% glucose enriched with [6,6‐^2^H_2_]‐glucose to maintain a P‐glucose of 5.5 mmol/L.

On the day of the HEC, fasting blood samples were obtained for analysis of plasma glucose, plasma free fatty acids (FAs), serum insulin, and serum C‐peptide.

### 2.3 Analytic procedures including measurement of sCD163

Blood collected in pre‐chilled EDTA tubes (for analysis of FAs) was immediately centrifuged, while clot‐activator tubes (for insulin and C‐peptide) were left for 30 min before centrifugation. Eppendorf tubes containing EDTA were immediately centrifuged and used for analysis of P‐glucose using YSI model 2300 STAT plus (YSI). Plasma FAs (NEFA C kit; Wako Chemicals GmbH) were measured using enzymatic colorimetric methods (Hitachi 912 automatic analyzer; Boehringer), while serum C‐peptide and insulin were analyzed using AutoDELFIA fluoroimmunoassay (Wallac OY). HbA1c was measured using high‐pressure liquid chromatography (Tosoh Bioscience).

Measurements of sCD163, ferritin, high‐sensitivity C‐reactive protein (hs‐CRP), interleukin‐6 (IL‐6), monocyte chemoattractant protein‐1 (MCP‐1), and tumor necrosis factor‐α (TNF‐a) were performed at the Department of Clinical Biochemistry, Aarhus University Hospital, Aarhus, Denmark in plasma samples that had been frozen at −80°C immediately after collection.

Plasma sCD163 was determined in duplicate by an in‐house sandwich ELISA using a BEP‐2000 ELISA‐analyzer (Dade Behring, Dade Behring) essentially as previously described (Moller et al., 2002). Control samples and plasma standards ranging from 6.25 to 200 μg/L were included in each run to avoid bias. The limit of detection was 6.25 μg/L. sCD163 is resistant to repeated rounds of freezing and thawing (Moller et al., 2002). We have previously established a reference intervals for sCD163 (0.69–3.86 mg/L) in a large cohort of healthy individuals using the same assay (Moller, 2012). MCP‐1 and TNF‐α were measured by electrochemiluminescence on a MESO QuickPlex SQ 120 instrument using U‐Plex kits (Meso Scale Discovery [MSD]). Ferritin was measured by electrochemiluminescence on an ADVIA Centaur XPT Immunoassay System (Siemens) and hs‐CRP by turbidimetry on an ADVIA Chemistry XPT System (Siemens).

### 2.4 Calculations and statistical analysis

Rate of appearance (Ra) and disappearance (Rd) of glucose were calculated from the last 30 min of the basal and clamp periods using Steele's equation (Steele, 1959). C‐peptide concentration in the basal period was used to assess basal hepatic IS (HISI_basal_ = 10^6^/[Ra_basal_ x C‐peptide_basal_]). Muscle IS was estimated as the tracer‐determined rate of disappearance (Rd) of glucose during the last 30 min of the HEC calculated by use of non‐steady‐state equations and expressed as milligrams per minute per kilograms of fat‐free mass (ffm). Correction for clamp insulin concentration was performed by dividing Rd with the mean serum insulin concentration disposal (Rd_clamp_/insulin_clamp_) to calculate the ultimate estimate of muscle IS (Bojsen‐Moller et al., 2014). Adipose tissue IS was assessed as the FA suppression by insulin, that is, the difference between basal and clamp FA concentrations expressed as a percentage of the basal concentration.

We calculated the Homeostasis Model Assessment 2 of Insulin Resistance (HOMA2‐IR) based on fasting glucose and C‐peptide using the online tool (https://www.dtu.ox.ac.uk/homacalculator/).

One‐way Analysis of Variance (ANOVA) was used for the comparison of multiple groups, and Student's *t*‐test for normally distributed variables between the groups. For non‐normally distributed variables, Kruskall–Wallis, and Mann–Whitney tests, respectively, were used. The relationship between sCD163 and other continuous variables was analyzed by simple linear regression.

Postoperative changes were analyzed by ANOVA in a linear mixed‐effects model using time from surgery and group as fixed effects and individual subjects as random effect.

All data are expressed as means ± standard error of mean (SEM) or proportions, and *p*‐values ≤0.05 were considered statistically significant. STATA version 14.0 ^®^StataCorp LP was used for data analysis.

## 3 RESULTS

### 3.1 Patient characteristics and effects of RYGB

The characteristics of patients at baseline and after RYGB, as well as the early and late effects of RYGB on hepatic and muscle IS have been presented earlier (Albers et al., 2015; Bojsen‐Moller et al., 2014, 2017) and are recapitulated in Table 1. In brief, both patients with preoperative T2D and patients with normal glucose tolerance lost weight following the procedure, accompanied by early improvements in endogenous glucose production and hepatic IS, followed by later improvements in adipose tissue and muscle IS.

**TABLE 1:** Patient characteristics at baseline and after RYGB in patients with diabetes and normal glucose tolerance before and 1 week, 3 months, and 1 year after RYGB

	Diabetes				NGT				Mixed‐effect model ANOVA		
	Before	1‐week	3 months	1‐year	Before	1‐week	3 months	1‐year	Time	Group	Time x Group
Male : female	4 : 6	4 : 4	4 : 6	4 : 5	3 : 7	3 : 5	3 : 7	3 : 6	—	—	—
Age (years)	43 ± 10				40 ± 9				—	0.43	—
BMI (kg/m^2^)	38.9 ± 1.6	37.3 ± 1.7	33.1 ± 1.5	30.8 ± 1.7	40.2 ± 0.8	37.9 ± 0.9	33.2 ± 1.1	28.5 ± 1.5	<0.01	0.48	<0.01
Weight (kg)	121 ± 9	118 ± 9	103 ± 8	96 ± 8	117 ± 5	112 ± 6	97 ± 5	83 ± 5	<0.01	0.52	0.06
sCD163 (mg/L)	2.7 ± 0.4	2.3 ± 0.2	2.1 ± 0.3	1.9 ± 0.1	2.2 ± 0.2	2.0 ± 0.2	1.8 ± 0.2	1.9 ± 0.2	0.01	0.18	0.08
ALT (IU/L)	35 ± 9	55 ± 8	33 ± 7	35 ± 6	27 ± 3	52 ± 15	21 ± 4	32 ± 11	<0.01	0.44	0.86
Fasting glucose (mmol/L)	8.7 ± 0.5	6.6 ± 0.4	5.7 ± 0.3	5.3 ± 0.1	5.1 ± 0.2	4.7 ± 0.2	4.8 ± 0.1	4.8 ± 0.04	0.45	<0.01	<0.01
HbA1c (mmol/mol)	53 ± 3.3	—	41 ± 2.2	39 ± 2.2	36 ± 1.1	—	34 ± 1.1	34 ± 1.1	<0.01	<0.01	<0.01
Basal Ra (mg/min)	207 ± 15	174 ± 11	176 ± 14	170 ± 11	175 ± 6	157 ± 9	166 ± 12	163 ± 8	<0.01	0.26	0.22
HISI	4.8 ± 1.0	7.7 ± 2.8	9.0 ± 1.9	8.7 ± 1.6	6.0 ± 0.4	9.2 ± 1.2	11.9 ± 1.2	12.6 ± 1.1	<0.01	0.16	0.39
Rd/I_ffm_ (mg/kg_ffm_/min per pmol/L)	14.0 ± 2.2	16.2 ± 3.0	24.7 ± 3.1	25.8 ± 4.0	22.1 ± 3.1	18.0 ± 2.0	30.5 ± 2.9	38.7 ± 6.5	<0.01	0.07	0.08
Fasting fatty acids (μmol/L)	705 ± 56	841 ± 36	740 ± 52	519 ± 31	635 ± 67	787 ± 41	670 ± 50	567 ± 71	<0.01	0.34	0.37
Suppression of fatty acids (%) during HEC	80 ± 3	69 ± 4	89 ± 2	95 ± 1	86 ± 2	86 ± 3	93 ± 1	95 ± 1	<0.01	0.07	<0.01
Fasting triglycerides (mmol/L)	2.3 ± 0.7	1.8 ± 0.3	1.4 ± 0.4	1.1 ± 0.2	1.2 ± 0.1	1.4 ± 0.1	1.0 ± 0.1	1.2 ± 0.2	0.61	0.01	0.04
Fasting adiponectin (μg/ml)	5.1 ± 0.7	4.4 ± 0.9	5.9 ± 0.9	8.5 ± 1.5	6.4 ± 0.7	5.6 ± 0.8	8.4 ± 1.0	12.5 ± 1.4	<0.01	0.28	0.02

Note

Parameters are presented as means ± standard error of mean and as total number for categorical variables.

Abbreviations: ALT, alanine transaminase; BMI, body mass index; HEC, hyperinsulinemic‐euglycemic clamp; HISI, hepatic insulin sensitivity; NGT, normal glucose tolerance; Ra, rate of appearance of glucose; Rd/I_ffm_, glucose disposal adjusted for clamp insulin concentration and fat‐free mass (muscle insulin sensitivity); sCD163, soluble CD163.

### 3.2 Macrophage activation at baseline and after RYGB

**FIGURE 1:**
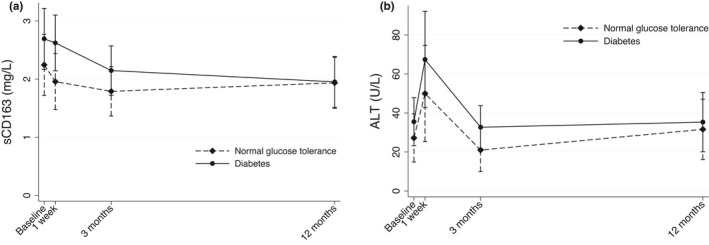
Concentrations of sCD163 and ALT in patients with preoperative diabetes and normal glucose tolerance at baseline and after RYGB. Means with 95% confidence intervals. (a) sCD163, time x group *p* = 0.08; (b) ALT, time x group *p* = 0.86. Analyzed using ANOVA in a linear mixed‐effects model using time from surgery and group as fixed effects and individual subjects as random effect

Preoperative concentrations of sCD163 were numerically but not significantly higher in patients with diabetes compared with patients with NGT (2.7 ± 0.4 vs. 2.2 ± 0.2 mg/L, *p* = 0.2). Following RYGB, sCD163 declined already at 1‐week postoperatively, with a further decrease during the subsequent 3 months and with more subtle changes within the remaining 9‐month follow‐up (Table 1, Figure 1a). The decrease in sCD163 after RYGB was overall more pronounced and consistent in patients with diabetes compared with subjects with NGT (*p* = 0.08), but the concentrations of sCD163 in the two groups were similar after 12 months of follow‐up (Table 1, Figure 1a).

### 3.3 Macrophage activation in relation to liver enzymes and IS

ALT was numerically but not significantly higher in patients with diabetes compared with patients with NGT before surgery (33 ± 23 vs. 21 ± 13 U/L, *p* = 0.18). Following RYGB, mean ALT increased within the first week and then declined back to preoperative concentrations at 3 months; this pattern was similar in the two groups (Figure 1b, *p* = 0.26). ALT showed little change during the rest of the follow‐up to 12 months.

**FIGURE 2:**
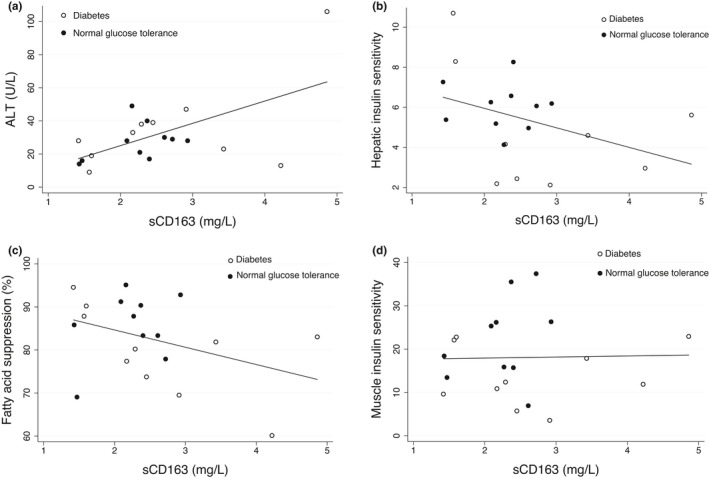
Preoperative associations of sCD163 with ALT, hepatic, adipose tissue, and muscle insulin sensitivity in patients with diabetes and normal glucose tolerance. (a) ALT, *r* = 0.58, *p* = 0.007; (b) hepatic insulin sensitivity, *r* = −0.39, *p* = 0.1; (c) adipose tissue insulin sensitivity expressed by fatty acid suppression, *r* = −0.39, *p* = 0.09; (d) muscle insulin sensitivity, *r* = 0.02, *p* = 0.96

**FIGURE 3:**
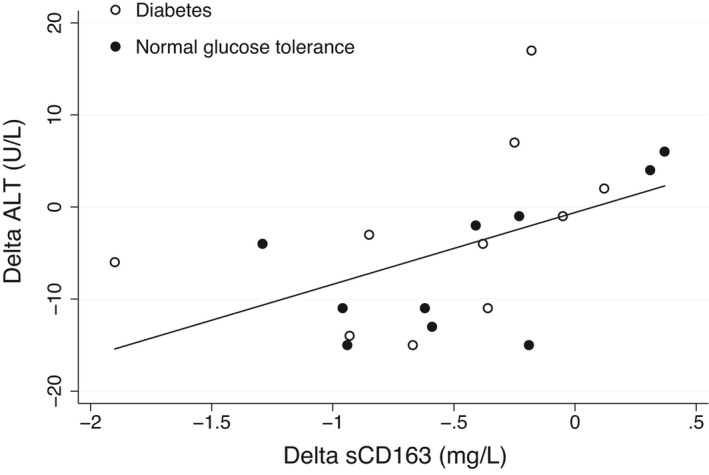
Association between the change in sCD163 within 1‐week after RYGB with the corresponding change in ALT. *r* = 0.53, *p* = 0.04

Before RYGB, sCD163 correlated with ALT (*r* = 0.58, *p* = 0.007, Figure 2a) and tended to be inversely associated with hepatic (*r* = −0.39, *p* = 0.1, Figure 2b) and adipose tissue IS (*r* = −0.39, *p* = 0.09, Figure 2c). There was no association between preoperative sCD163 and muscle IS (*r* = 0.02, *p* = 0.96, Figure 2d). Within the first week following RYGB, the change in sCD163 correlated significantly and positively with the change in ALT (*r* = 0.53, *p* = 0.04) despite the median decrease in sCD163 and simultaneous increase in ALT, that is, the patients with the largest decrease in sCD163 experienced the lowest ALT increase, whereas the few patients with an increasing sCD163 had the largest increase in ALT (Figure 3). The change in sCD163 during the first postoperative week showed no significant associations with the corresponding changes in hepatic (*r* = 0.31, *p* = 0.28), adipose tissue (*r* = −0.12, *p* = 0.68), or muscle IS (*r* = 0.41, *p* = 0.13).

**FIGURE 4:**
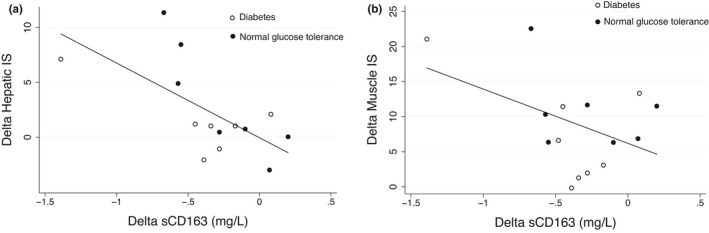
Association between the change in sCD163 from 1 to 12 weeks after RYGB with the corresponding changes in hepatic and muscle insulin sensitivity. (a) hepatic insulin sensitivity, *r* = −0.65, *p* = 0.01; (b) muscle insulin sensitivity. *r* = −0.45, *p* = 0.09

The decrease in sCD163 from 1‐week to 3 months post‐RYGB showed a significant inverse association with the improvement in hepatic IS (*r* = −0.65, *p* = 0.01, Figure 4a), and an inverse trend for muscle IS (*r* = −0.45, *p* = 0.09, Figure 4b) but was not significantly associated with the changes in ALT (*r* = −0.30, *p* = 0.28) or adipose tissue IS (*r* = −0.37, *p* = 0.17). At 3 months, sCD163 concentrations were significantly associated with ALT (*r* = 0.75, *p*<0.001) and showed an inverse trend for correlation with hepatic IS (*r* = −0.39, *p* = 0.1), but not with adipose tissue (*r* = −0.01, *p* = 0.98) or muscle IS (*r* = −0.07, *p* = 0.76).

At the end of follow‐up 12 months after RYGB, sCD163 concentrations correlated with ALT (*r* = 0.61, *p* = 0.007), but not with hepatic (*r* = −0.12, *p* = 0.64), adipose tissue (*r* = −0.02, *p* = 0.94), or muscle IS (*r* = 0.17, *p* = 0.51). The change in sCD163 from 3 to 12 months was not significantly associated with the corresponding changes in ALT, hepatic, adipose tissue, or muscle IS.

There were no significant associations between sCD163 and basal endogenous glucose production (basal Ra) or the changes in these measures at baseline or any time point during follow‐up.

sCD163 correlated significantly with HOMA2‐IR at baseline (*r* = 0.49, *p* = 0.048) and after 1‐week (*r* = 0.79, *p* = 0.001). The change in sCD163 during the first 3 months of post‐RYGB was associated with the corresponding change in HOMA2‐IR (*r* = 0.63, *p* = 0.006).

### 3.4 Associations of macrophage activation with free fatty acids, adiponectin, and lipid levels

sCD163 did not show significant associations with the concentrations or changes of FAs or triglycerides in the fasting state at any time point or interval during the study period. We observed an inverse association between sCD163 and adiponectin preoperatively (*r* = −0.46, *p* = 0.04), and trends to associations at 1‐week (*r* = −0.45, *p* = 0.09) and 3 months (*r* = −0.38, *p* = 0.1), but not 12 months after RYGB. The changes in sCD163 did not correlate with the corresponding changes in adiponectin.

The change in sCD163 during the first 3 months after RYGB was positively associated with the corresponding change in triglycerides (*r* = 0.47, *p* = 0.04) and inversely with the change in high‐density lipoprotein (HDL) (*r* = −0.59, *p* = 0.008). There were no significant associations between sCD163 and HDL or triglycerides at any of the other time points, and low‐density lipoprotein at any time points.

### 3.5 Inflammatory markers

The levels of ferritin, hs‐CRP, IL‐6, MCP‐1, and TNF‐α are shown in Table 2. Ferritin increased in the first week after RYGB, with a consistent decrease for the rest of the follow‐up; hs‐CRP and IL‐6 showed a similar pattern over time. MCP‐1 had an overall decrease, whereas TNF‐α did not change significantly over the course of the study (Table 2).

**TABLE 2:** Markers of inflammation at baseline and after RYGB in patients with diabetes and normal glucose tolerance before and 1‐week, 3 months, and 1‐year after RYGB

	Diabetes				NGT				Mixed effect model ANOVA		
	Before	1‐week	3 months	1‐year	Before	1‐week	3 months	1‐year	Time	Group	Time x Group
Ferritin (µg/L)	144 ± 27	219 ± 55	81 ± 19	50 ± 14	77 ± 24	121 ± 28	41 ± 12	30 ± 8	<0.01	0.04	0.07
hs‐CRP (mg/L)	7.8 ± 2.6	21.9 ± 4.9	4.8 ± 1.3	1.3 ± 0.7	4.4 ± 0.9	27.2 ± 9.1	3.2 ± 0.7	1.3 ± 0.5	<0.01	0.39	0.51
IL‐6 (ng/L)	5.4 ± 0.8	5.9 ± 0.5	4.8 ± 0.4	3.8 ± 1.6	5.3 ± 0.6	8.5 ± 1.5	4.8 ± 0.3	7.3 ± 3.5	0.16	0.93	0.34
MCP‐1 (pg/ml)	280 ± 26	302 ± 31	260 ± 32	254 ± 29	315 ± 43	264 ± 18	319 ± 57	248 ± 23	0.01	0.46	0.20
TNF‐α (pg/ml)	1.7 ± 0.2	2.0 ± 0.3	1.8 ± 0.1	1.4 ± 0.2	1.8 ± 0.2	1.9 ± 0.2	1.9 ± 0.1	1.7 ± 0.1	0.16	0.53	0.58

Note

Parameters are presented as means ± standard error of mean.

Abbreviations: Hs‐CRP, high sensitivity C‐reactive protein; IL‐6, interleukin 6; MCP‐1, monocyte chemoattractant protein‐1; NGT, normal glucose tolerance; TNF‐α, tumor necrosis factor‐α.

We observed no significant associations between sCD163 and ferritin, IL‐6, MCP‐1, or TNF‐a. Baseline sCD163 was associated with hs‐CRP (*r* = 0.70, *p* < 0.001), and the change in sCD163 from 1‐week to 3 months post‐RYGB tended to correlate with the corresponding change in hs‐CRP (*r* = 0.44, *p* = 0.07).

Baseline hs‐CRP showed an inverse trend to associate with hepatic IS (*r* = −0.41, *p* = 0.08). Ferritin was inversely associated with hepatic IS at baseline (*r* = −0.57, *p* = 0.01), 1‐week (*r* = −0.55, *p* = 0.04), and 12 months (r = −0.49, *p* = 0.046) after RYGB. MCP‐1, TNF‐a, and IL‐6 were not associated with hepatic IS.

Ferritin correlated inversely with muscle IS at baseline (*r* = −0.43, *p* = 0.06), after 1‐week (*r* = −0.59, *p* = 0.02), 3 months (*r* = −0.49, *p* = 0.03), and 12 months (*r* = −0.50, *p* = 0.03) post‐RYGB. Furthermore, the change in ferritin from baseline to 1‐week (*r* = −0.66, *p* = 0.007) and from 1‐week to 3 months (*r* = −0.45, *p* = 0.09) was inversely associated with the corresponding changes in muscle IS. The other inflammatory markers showed no association with hepatic IS.

Hs‐CRP correlated inversely with adipose tissue IS at baseline (*r* = −0.45, *p* = 0.04), but not at other time points. None of the other markers of inflammation were associated with adipose tissue IS.

## 4 DISCUSSION

The main findings of our study are the associations between the degree of macrophage activation assessed by sCD163 and liver injury measured by ALT and hepatic IS assessed by state‐of‐the‐art methods in severely obese patients. Further, we demonstrated a decrease in sCD163 in association with ALT levels and inversely with hepatic IS especially in the early phase after RYGB, whereas the association with muscle IS was not significant. After 12 months, the concentrations of sCD163 and ALT were still significantly and directly correlated, whereas no associations between sCD163 and IS were found.

The levels of sCD163 in our patients were within the range established in healthy subjects (Moller, 2012). This is consistent with our previous findings in patients with severe obesity and MAFLD, with low sCD163 levels except in patients with cirrhosis (Kazankov et al., 2015, 2016), who are not common in cohorts of patients undergoing bariatric surgery (Kazankov et al., 2015; Mueller et al., 2015). Nevertheless, in the previous reports sCD163 correlated significantly with liver inflammation and fibrosis, as well as insulin resistance, which was also the case in the present study and suggests that macrophages play a role in low‐grade inflammation associated with MAFLD and insulin resistance.

Notably, the association between the changes in sCD163 and hepatic IS was not significant within the first week after RYGB. We observed a spike in ALT levels during this period reflecting the transient liver injury attributed to the surgical procedure (Tan et al., 2003). Furthermore, during the first week after surgery with a very low caloric intake a significant load of free fatty acids is delivered to the liver because of enhanced lipolysis in the peripheral and visceral adipose tissues, leading to hepatocyte toxicity due to accumulation of intermediary metabolites and increased reactive oxygen species, which could be a mechanistic link to the increase in ALT (Bojsen‐Moller et al., 2014; Koliaki et al., 2015). We cannot exclude that a reduction in subcutaneous and visceral fat and adipose tissue inflammation may have lessened the activation of macrophages contributing to the decrease in sCD163 concentrations, and since patients with the largest decrease in sCD163 experienced the lowest ALT increase, it could be speculated that those with a more favorable phenotype and less inflammation in adipose tissues had a lower flux of fatty acids and cytokines to the liver; however, proving this notion requires further investigation. Taken together, the first week post‐surgery may not be representative of hepatic events regarding macrophage activation and glucose metabolism. Similarly, sCD163 did not correlate significantly with hepatic IS at the end of follow‐up 12 months after surgery, possibly explained by the subtle changes in sCD163 and hepatic IS in the last 9 months of follow‐up.

In contrast, decreasing sCD163 levels correlated strongly with continuous improvement in hepatic IS during the first 3 months of follow‐up when the immediate effects of surgery‐related mechanical stress had subsided, and sCD163 was associated with ALT and inversely with hepatic IS at the 3‐month visit. This may be reflective of the association between macrophages and steatosis established in experimental models, where macrophages were able to induce steatosis (Obstfeld et al., 2010), and in turn, steatotic and lipotoxic hepatocytes contributed to macrophage activation (Pan et al., 2015; Tomita et al., 2016). Likewise, the relationship between macrophages and steatosis was demonstrated in obese patients by Fjeldborg et al. who found a dynamic correlation between sCD163 levels and the reduction in intrahepatic lipid content by MR spectroscopy following RYGB (Fjeldborg et al., 2015). On the other hand, the association between steatosis and IS impairment is also well‐established (Yki‐Jarvinen, 2014). Obviously, sCD163 may originate from macrophages both in the liver and adipose tissues, and both fat mass as well as inflammation in the liver and adipose tissue was greatly reduced 12 months after RYGB (Pedersen et al., 2020). However, we have previously shown a sCD163 gradient across the liver in morbidly obese patients supportive of a considerable production by hepatic macrophages (Kazankov et al., 2015). Thus, liver macrophages may be one of the factors linking the alleviation of liver steatosis and hepatocyte injury with improved hepatic IS after bariatric surgery.

The observed associations of sCD163 support the idea of a link between macrophages and impaired hepatic IS. Naturally, correlation is not equivalent to causality, and a case could be made for macrophage activation by metabolic derangement and hyperinsulinemia, as insulin may trigger an inflammatory response in macrophages (Iida et al., 2001) as well as induce their infiltration in adipose tissue with detrimental effects on hepatic and muscle IS (Mauer et al., 2010). However, direct contribution of macrophages to hepatic IS impairment has been repeatedly reported. Thus, hepatic IS was improved by silencing of the pro‐inflammatory mediator NF‐κB specifically in the liver‐resident Kupffer cells of high‐fat diet (HFD)‐fed mice (Tencerova et al., 2015). Furthermore, macrophage depletion showed protective effects against hepatic steatosis and IR, while pro‐inflammatory macrophages decreased hepatocyte responsiveness to insulin in vitro (Huang et al., 2010; Lanthier et al., 2010). Soluble CD163 has previously been associated with HOMA‐IR, a surrogate measure of hepatic IR, in patients with diabetes as well as obese and normal‐weight subjects (Parkner et al., 2012; Zanni et al., 2012). Likewise, in a recent study of a large number of patients at risk of type 2 diabetes, repeated sCD163 measurements correlated with HOMA‐IR and were predictive of incident dysglycemia (Semnani‐Azad et al., 2020). Taken together, we believe that our results support and expand the notion of macrophages as key players in impaired hepatic IS in obesity.

In our study, sCD163 showed only a preoperative inverse correlation with adipose tissue IS as well as weak and inconsistent associations with muscle IS, which contrasts a previous study by Kracmerova et al. who showed an association of sCD163 with peripheral IS in a heterogeneous cohort of nondiabetic obese women, whereas the association with hepatic IS was not reported (Kracmerova et al., 2014). Similarly, our group recently demonstrated a significant association between sCD163 and adipose tissue IS, but not hepatic IS in nondiabetic patients with biopsy‐proven MAFLD (Rosso et al., 2019). Importantly, the majority of them were not obese as opposed to our present cohort of severely obese patients undergoing bariatric surgery, and we believe that the variation in associations of sCD163 with hepatic and muscle IS may be explained by the different study populations. Indeed, in morbidly obese and especially diabetic patients with more pronounced endogenous glucose production, macrophages may have a greater effect on hepatocytes leading to impaired hepatic IS. In this view, it is notable that in our study sCD163 was not associated with the basal concentrations of free fatty acids contrary to nondiabetic and less obese patients (Rosso et al., 2019), which suggests that local intrahepatic events may influence liver macrophage activation to a higher degree than factors produced in the adipose tissue in the setting of morbid obesity, although the observed association with adiponectin indicates at least some degree of cross‐talk with fat depots. Interestingly, in mice fed a high‐fat and high‐cholesterol diet, the expression of genes associated with macrophage recruitment and inflammation in adipose tissue preceded hepatic expression (Stanton et al., 2011), which may suggest a prominent role of adipose tissue macrophages in moderate metabolic derangement, with hepatic macrophages being more important at a later stage such as severe obesity.

In our study, sCD163 showed no associations with inflammatory markers except a weak association with hs‐CRP, although macrophages are known to produce cytokines such as TNF‐ a and IL‐6 (Kazankov et al., 2019). Furthermore, these markers did not consistently correlate with hepatic, muscle, or adipose tissue IS. Obviously, the limited sample size may provide an explanation for the lack of associations, however, it may also reflect the pleiotropic effects of macrophages in liver homeostasis and disease including MAFLD, with macrophages exerting their actions on liver inflammation and hepatic insulin resistance through multiple molecules and events not necessarily involving signals from cytokines.(Kazankov et al., 2019).

Our study has strengths and limitations. The most important strength was the prospective design of the study with a complete and detailed 12‐month follow‐up of the patients. Furthermore, all patients were well classified and comprehensively investigated using robust well‐established and validated methods including repeated HEC with glucose tracer, a state‐of‐the‐art tool for the assessment of hepatic and muscle IS. The most significant limitation was the lack of determination of CD163 expression on hepatic and adipose tissue macrophages for a more complete evaluation of macrophage function. Another potential drawback was the low number of patients in the study, which may have an impact on the analysis of associations.

In conclusion, we demonstrated cross‐sectional and dynamic associations of macrophage activation with hepatic IS to a higher degree than muscle IS in morbidly obese patients during the first 3 months following RYGB, which contrasts the data derived from nonobese and nondiabetic patients. This may suggest for the first time that macrophages are differently involved in the development of IR in severe obesity compared to nonobese and nondiabetic patients providing new insights to the role of macrophages in MAFLD and obesity and the associated insulin resistance.

## CONFLICT OF INTEREST

No conflict.

## AUTHOR CONTRIBUTIONS

Konstantin Kazankov: study design and conception, data analysis and interpretation, manuscript preparation; Kirstine Nyvold Bojsen‐Møller: study design and conception, study subject inclusion, data acquisition and interpretation, critical revision of the manuscript for intellectual content; Holger Jon Møller: data analysis and interpretation, critical revision of the manuscript for intellectual content; Sten Madsbad: study design and conception, administrative support, data interpretation, critical revision of the manuscript for intellectual content; Henning Grønbæk: study design and conception, data interpretation, study supervision, critical revision of the manuscript for intellectual content. All authors have approved the submitted manuscript.
